# Bio-Rad’s Bio-Plex® suspension array system, xMAP technology overview

**DOI:** 10.3109/13813455.2012.705301

**Published:** 2012-08-02

**Authors:** Brett Houser

**Affiliations:** Bio-Rad Laboratories, Inc., Hercules, California, USA

**Keywords:** Immunoassay, multiplex detection, ELISA, Bio-Plex®, xMAP, Luminex

## Abstract

The Bio-Plex® system utilizes xMAP technology to permit the multiplexing of up to 100 different analytes. Multiplex analysis gives researchers the ability to look at analytes simultaneously providing more information from less sample volume in less time than traditional immunoassay methods. Similar to ELISA, xMAP utilizes an antibody sandwich for detection but differs from ELISA in capture substrate and detection method. Rather than a flat surface, Bio-Plex®assays make use of differentially detectable bead sets as a substrate capturing analytes in solution and employs fluorescent methods for detection. These bead sets identify the analytes and detection antibodies are used to measure the quantity of analyte. The use of differentially detectable beads enables the simultaneous identification and quantification of many analytes in the same sample.

## Background

For more than 50 years, immunoassays have allowed sensitive and highly specific detection of analytes of interest in biological samples in life science research and clinical diagnostics. Immunoassays provide informa-tion to researchers on the roles proteins and other bio-molecules play in a myriad of biological processes and diseases. The first immunoassay was developed by Yalow and Berson (1960), who received the Nobel Prize for their efforts to measure insulin levels. These initial assays used radiolabels for detection. The radioimmunoassay (RIA) would remain the standard for detection of bio-analytes for more than 10 years because of its extraordinary sen-sitivity, in spite of the health risks and disposal issues posed by the use of radioisotopes. The search for a suit-able alternative to the RIA led to the development of the enzyme-linked immunosorbent assay (ELISA) in the early 1970s (Engvall and Perlmann 1971, Van Weeman and Schuurs 1971). ELISA uses an enzymatic reaction as the basis of detection, rather than a radioactive sig-nal. While early versions did not rival the sensitivity of the RIA, the development of highly specific monoclonal antibodies and chemiluminescence detection resulted in ELISA assays with sensitivity that exceeds that of radiola-bels. Today, key advantages of ELISA are its ease of use, flexibility and low cost. The impact of immunoassays on life science research and clinical diagnostics has been enormous, with almost 10,000 studies published per year that include the terms “enzyme immunoassay” and “enzyme-linked immunoassay” (Lequin 2005).

## Multiplex detection and measurement using xMAP technology

**Figure 1:**
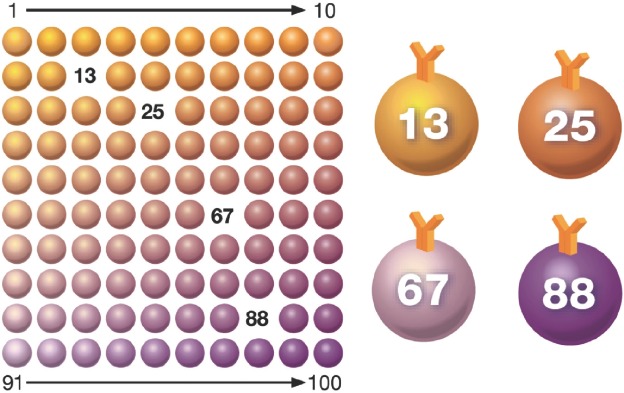
Multiplex immunoassay technology. Beads are colored internally with two different fluorescent dyes (red and infrared). Ten different concentrations of red and infrared dyes are used to generate 100 distinct bead regions. Each bead region is conjugated to a specific target analyte.

The Bio-Plex® suspension array system utilizes xMAP technology, licensed from Luminex Corp., to permit the multiplexing of up to 100 different assays within a single sample. This technique involves 100 distinctly coloured bead sets created by the use of two fluorescent dyes at distinct ratios. Beads are conjugated with a reagent specific to a particular bioassay. The reagents may include antigens, antibodies, oligonucleotides, enzyme substrates or receptors. The technology makes use of multiple assays whereby one antibody to a specific analyte is attached to a set of beads with the same colour and the second antibody is used to quantify the bound antigen. The use of different coloured beads enables the simultaneous detection of many other analytes in the same sample. Imaging or laser excitation is then used to determine the different assays by bead colours, and determine analyte concentration by measuring the reporter dye fluorescence (Figure 1).

**Figure 2:**
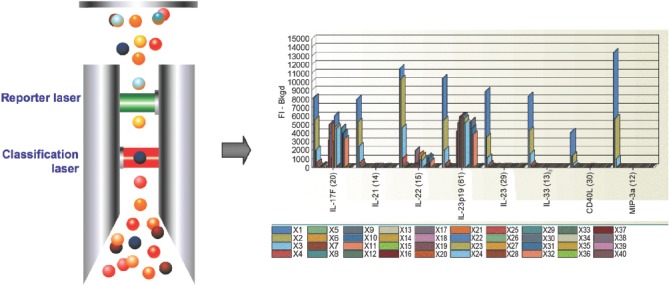
Data acquisition and reduction. Dyed beads are pushed through a detection chamber in a single file. The red classification laser (635 nm) interrogates internal dye to identify bead regions. The green reporter laser (532 nm) interrogates fluorescent reporter to measure analyte concentration.

For laser excitation detection (Bio-Plex 200 and Bio-Plex 3D) the contents of each microplate well are drawn into the array reader and precision fluidics align the beads in single file through a flow cell, where two lasers excite the beads individually. The red classification laser excites the dyes in each bead, identifying its spectral address. The green reporter laser excites the reporter molecule associated with the bead, which allows quan-titation of the captured analyte. High-speed digital signal processors and software record the fluorescent signals simultaneously for each bead, translating the signals into data for each bead-based assay (Figure 2).

**Figure 3:**
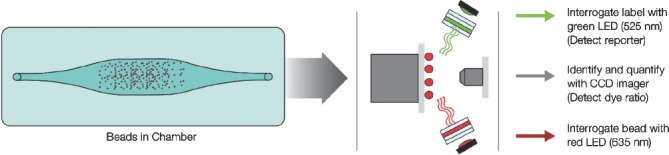
Immobilized magnetic beads illuminated with LED’s and imaged using CCD technology.

The Bio-Plex MAGPIX system employs low-cost light-emitting diodes (LEDs) and a charge-coupled device (CCD) imager to illuminate and image immobilized magnetic beads Figure 3. Unlike flow-based systems that quantitate bead events individually, the Bio-Plex MAGPIX system reads all of the beads at once. This dependable design is not only very robust, but also reduces instrument preparation time compared to flow-based design–all the while giving very comparable data.

## Workflow overview

**Figure 4:**
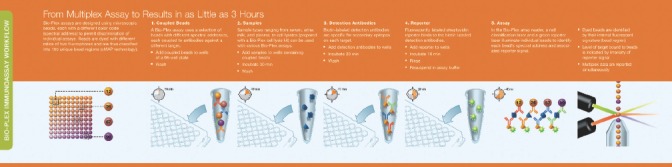
xMAP workflow.

Similar to ELISA, a majority of assays are designed accord-ing to a capture sandwich immunoassay format. Briefly, the capture antibody-coupled beads are first incubated with antigen standards or samples for a specific time. The plate is washed to remove unbound materials, followed by incu-bation with biotinylated detection antibodies. After wash-ing away the unbound biotinylated antibodies, the beads are incubated with a reporter streptavidin-phycoerythrin conjugate (SA-PE). Following removal of excess SA-PE, the beads are passed through the array reader, which measures the fluorescence of the bound SA-PE (Figure 4).

The substrate for the antibody sandwich is the bead. Bead characteristics define instrument compatibility and workflow and can be classified into two categories, non-magnetic and magnetic. The non-magnetic beads are smaller in size (5.6 μm and are used with the vacuum workflow; they are not compatible with instruments that utilize magnets for imaging purposes. Non-magnetic beads require filter plates and vacuum filtration to wash the beads. Magnetic beads are coated with magnetite and are therefore larger in size (6.5 μm); they can be used with the magnetic workflow as well as vacuum workflows. These magnetic beads are compatible with all currently available life science instruments from any Luminex partner. In the magnetic workflow, the beads are washed in the well with dispense and aspiration washing.

## About the beads

**Figure 5:**
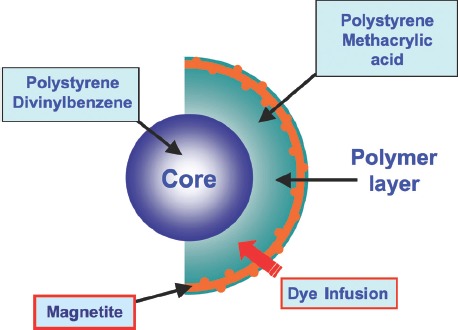
Magnetic bead architecture.

xMAP assays may contain non-magnetic or magnetic beads as substrates. Magnetic COOH beads are the new-est core components of xMAP assays. They are unique in that they exhibit both fluorescent and magnetic properties. The beads are stained with a fluorescent dye formulation proprietary to Luminex (Figure 5). The stain-ing process involves swelling the bead particles in a dye containing solvent allowing the dye molecules to infuse the coating or the polymer layer. Removal of the solvent in a subsequent step shrinks the beads and traps the dye molecules within the bead particles.

**Figure 6:**
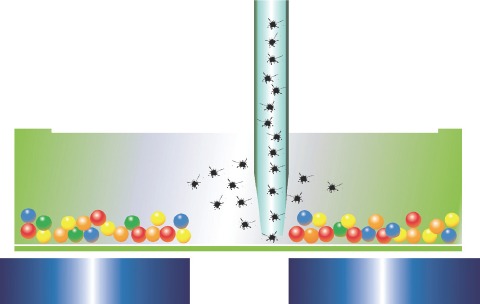
Hands-free washing and separation of magnetic beads.

The use of magnetic beads confers a number of benefits, including ease of separation and suitability for automation. Once coated with ligand molecules, the magnetic beads are useful for the capture and separation of a variety of targets. Unwanted sample constituents such as large or fibrous particulates or a viscous sample matrix may be washed away following a simple magnetic separation step. Overall, the highly efficient magnetic separation eliminates potentially interfering molecules, allowing sensitive detection of targets (Figure 6).

## Advantages of bead based multiplex detection over conventional methods

### ELISA

The xMAP technology is frequently compared to the traditional ELISA technique, which is limited by its ability to measure only a single antigen. The inability of traditional ELISA to multiplex presents additional limitations in sampling volume, cost and labour (Table 1). While the basic concepts of detection are similar between ELISA and xMAP technology, there are some important differences that centre on the capture antibody support. Unlike traditional ELISA, xMAP capture antibodies are covalently attached to a bead surface, effectively allowing for a greater surface area as well as a matrix or free solu-tion/liquid environment to react with the analytes. The suspended beads allow for assay flexibility in a singleplex or multiplex format.

**Table 1:** Side-by-side comparison: Analysing 27 cytokines in 80 samples.

	ELISA	Bio-Plex
Number of cytokines	27	27
Number of samples	80	80
Total data points	2160	2160
Number of 96-well plates	27	1
Data points per plate	80	2160
Total time required	>60 hours	3 hours
Sample volume	Serum or plasma, >1 ml *Cell culture supernatant, >1 ml*	Serum or plasma, 12.5 μ Cell culture supernatant, 50 μl
Assay range	3 to 4 logs dynamic range	5 to 6 logs dynamic range

Note: *Based on 50 μl/well sampling volume.

### Western blot

Bio-Plex cell signalling assays can be used in place of classic western blot methods. For example investigat-ing five proteins in 12 samples by western blot would require five gels and blots, 5 times more samples (125 ul vs. 25 ul) and about 5 hours longer to obtain results (Table 2).

**Table 2:** Side-by-side comparison: Analysing 5 phosphoproteins in 12 samples.

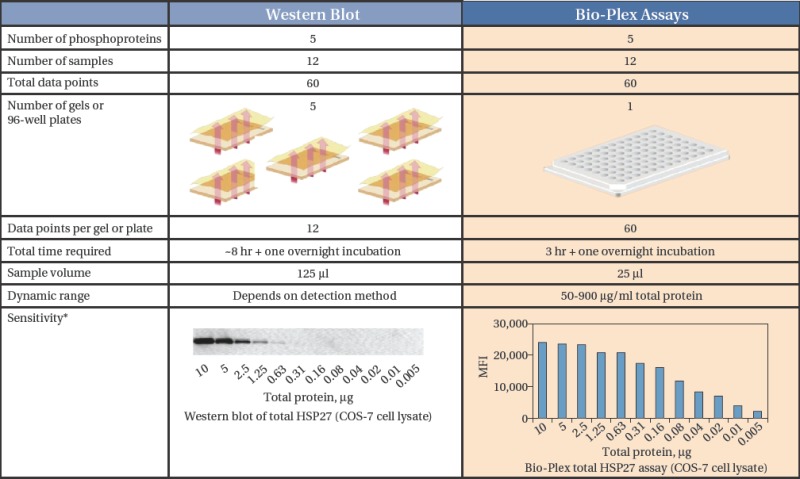

### Multiplex assay applications

The use of xMAP technology is well established with over 7000 peer-reviewed publications to date. Bio-Rad’s Bio-Plex®assays are available for many classes of bio-molecules and species including cytokines and growth factors, specialized disease state panels for cancer, acute phase, immune response and diabetes. Assays are avail-able in several flexible configurations. Commercially available kits are typically developed with optimized pro-tocols. Alternatively, tools and individual components are available for researchers to develop their own assays.

### Data acquisition and analysis via Bio-Plex® software

An integral component of multiplex immunoassay experiments, Bio-Plex® software provides an intui-tive interface for input of sample properties, allowing researchers to handle clinical information from large cohorts of samples and tools for analysis of complex pro-teomic data generated by the Bio-Plex® system.

**Figure 7:**
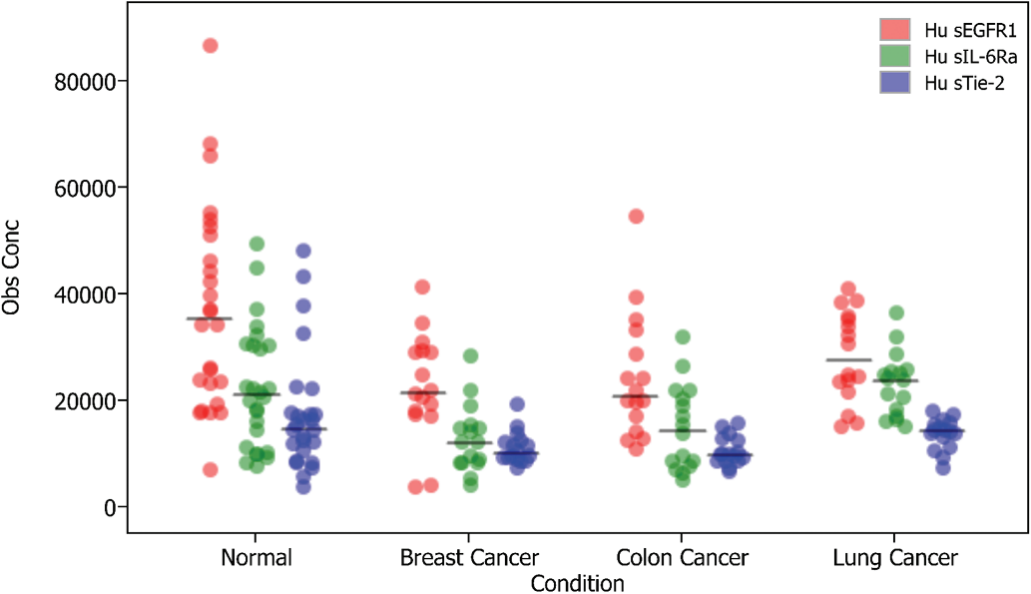
Visualization of sample relationships by analyte using Bio-Plex® Data Pro Software.

Biomarker discovery driven data analysis tools include multiple modes of spectral visualization. Differentially expressed proteins are sought through univariate analy-ses, including Mann-Whitney and Kruskal-Wallis tests, receiver operating characteristic curves, and dot plots. Relationships between samples may be further explored with Bio-Plex® Data Pro software (Figure 7).

### Definition of quality

Assay quality is defined by a series of experiments establishing performance characteristics of the assay, such as accuracy, precision, specificity and sensitivity. Taking into account that an assay should provide reliable results, the concept of assay quality is generally viewed in a broader perspective. Design of the assay workflow, the sample matrices to be tested, and the utility of the test results should also be taken into consideration.

Immunoassay standard curves are inherently nonlinear. As such, a minimum of six non-zero standard points assayed in duplicate is recommended to ensure the robustness of an assay. The concentration–response relationship is most often fitted to a 4- or 5-parameter logistic model.

Bio-Rad, Bio-Plex® multiplex immunoassays are subject to rigorous quality measures. The assays are developed for optimal performance and dynamic ranges that are relevant for investigating biological states.

Bio-Rad was first among the current life science partners to license this dynamic technology. The Bio-Plex® product family provides a complete solution: multiplex assays, the Luminex instruments, and proprietary innovative software for data generation and analysis.

Over the years, the power and ease-of-use of Bio-Plex® software has delivered dominant market share in xMAP instruments; as a result, most xMAP researchers use Bio-Plex® to get the answers they need.

## References

[b1] Engvall E, Perlmann P (1971). Enzyme-linked immunosorbent assay (ELISA). Quantitive assay of immunoglobin G. Immunochemistry.

[b2] Leguin RM (2005). Enzyme immunoassay (EIA)/enzyme-linked immunosorbent assay (ELISA). Clin Chem.

[b3] Van Weeman BK, Schuurs AH (1971). Immunoassay using antigenenzyme conjugates. FEBS Lett.

[b4] Yalow RS, Berson SA (1960). Immunoassay of endogenous plasma insulin in man. J Clin Invest.

